# Updated Incidence, Treatment and Survival of a Nationwide Cohort of Patients with Peritoneal Metastases of Unknown Origin

**DOI:** 10.1007/s13193-022-01567-x

**Published:** 2022-06-21

**Authors:** Anouk Rijken, Caroline Loef, Yes A. J. van de Wouw, Felice N. van Erning, Ignace H. J. T. de Hingh

**Affiliations:** 1 Department of Surgery, Catharina Cancer Institute, Eindhoven, Netherlands; 2 Department of Research and Development, Netherlands Comprehensive Cancer Organization, Utrecht, the Netherlands; 3 Department of Medical Oncology, VieCuri Hospital, Venlo/Venray, the Netherlands; 4 GROW- School for Oncology and Development Biology, Maastricht University, Maastricht, the Netherlands

**Keywords:** Peritoneal metastases, Cancer of unknown origin, Incidence, Histology, Treatment, Survival

## Abstract

The aim of this study was to investigate the incidence, treatment and survival of patients with peritoneal metastases (PM) of unknown origin. All Dutch patients diagnosed in 2017 and 2018 with PM of unknown origin (PM-CUP) were evaluated. Data were extracted from the Netherlands Cancer Registry (NCR). Patients with PM-CUP were categorized into the following histological subtypes: 1) adenocarcinoma, 2) mucinous adenocarcinoma, 3) carcinoid, 4) unspecified carcinoma and 5) other. Treatments were compared between the different histological subtypes in patients with PM-CUP. Overall survival (OS) was calculated using the Kaplan–Meier method for all patients with cancer of unknown origin and between histological subtypes in patients with PM-CUP. Significant differences in OS were assessed by using the log-rank test. In total, 3026 patients were diagnosed with cancer of unknown origin, 513 (17%) among them were diagnosed with PM-CUP. Most PM-CUP patients received best supportive care only (76%), whereas 22% received systemic treatment and 4% underwent metastasectomy. Median OS was 1.1 months for all patients with PM-CUP but varied from 0.6 months to 30.5 months depending on the underlying histology. In this study, PM-CUP were diagnosed in 17% of all patients with cancer of unknown primary and the reported survival in this cohort was extremely poor. Since survival differed among histological subtypes and recently more treatment options became available for a selected group of patients with peritoneal malignancies, it is of great importance to identify the histology of the metastases and whenever possible the primary tumor.

## Introduction

Peritoneal metastases (PM) are thought to be caused mainly by dissemination of tumor cells trough the abdominal cavity. As a result, relatively high incidences of PM are described from multiple primary intra-abdominal tumors such as colorectal, ovarian and gastric cancer [1–4]. However, PM may also be diagnosed in patients in whom the primary tumor site is unknown and remains unknown after initial workup [5, 6]. In approximately 3–5% of all patients diagnosed with metastatic cancer, the primary tumor location remains unknown [5–7]. In patients with metastases from an unknown origin, survival is generally poor [7, 8].

For long, PM were generally considered as incurable with only very few treatment options available. However, the amount of new treatment strategies for PM from a variety of primary tumors is currently expanding. Multimodal treatments such as cytoreductive surgery and hyperthermic intraperitoneal chemotherapy (CRS-HIPEC) in a selected group of patients with PM from colorectal, ovarian or gastric cancer, have revealed promising results on survival in several studies [9–11]. In case of more extensive intraperitoneal disease not amendable for complete cytoreduction, alternative treatment options such as systemic therapy or chemotherapy applied intraperitoneally by pressurized intraperitoneal aerosol chemotherapy (PIPAC) or intraperitoneally administered chemotherapy (INTERACT) are currently being investigated [12–16]. This evolution in treatment options emphasizes the value of determining the primary tumor location in patients with PM whenever possible.

In PM from an unknown origin (PM-CUP), the underlying tumor histology differs among patients [5, 7]. This is important as PM from different tumor histologies may result in a different biological behavior and therefore require other diagnostic tools and treatment strategies. Thus, a better understanding of different histological subtypes in patients with an unknown primary tumor is warranted and may contribute to a more suitable approach in these patients.

Therefore, the aim of this study is to determine the incidence, treatment and survival of patients with PM-CUP and to gain more insight into the different histological subtypes of these patients.

## Methods

### Data source

For this nationwide cohort study, data were extracted from the Netherlands Cancer Registry (NCR). Specially trained data managers of the NCR routinely collect data on patient, tumor and treatment characteristics from medical records. For the specification of primary tumor location, location of metastases and histologic characteristics, the International Classification of Disease for Oncology (ICD-O) was used. The NCR provided follow-up information on vital status, which was obtained by linking NCR data to the municipal administrative database in which all deaths and emigrated inhabitants of the Netherlands are registered. The latest linkage with the municipal administrative database for the present study was January 31, 2020. Since all data were anonymized, no ethics approval was obligated for this study.

### Study population

All patients diagnosed in 2017 and 2018 with cancer of unknown primary (*C80.9*) were screened for eligibility. Patients with PM-CUP were included for analyses. PM were defined according to the ICD-O (*C48.0 – C48.2, C48.8*). Patients with PM were subcategorized as follows: 1) isolated PM-CUP, which included all patients with only PM-CUP and 2) PM-CUP and systemic metastases, which included all patients with PM-CUP and concurrent other metastases. Patient and tumor characteristics included in this study are sex, age and histological subtype. The histology of the primary tumor was categorized into 1) adenocarcinoma (*8140, 8144, 8310, 8380, 8441*), 2) mucinous carcinoma (*8480, 8481*), 3) carcinoid (*8240, 8249*), 4) unspecified carcinoma (*8000, 8001, 8010, 8020, 8012, 8032, 8041, 8046, 8070*) and 5) other (*8490, 8680, 8801, 8803, 8936, 8980, 8246, 8244, 8013, 8120, 8315, 8720*). The treatments for patients with PM-CUP were defined as: 1) metastasectomy, 2) systemic treatment or 3) only best supportive care (BSC) and no tumor directed treatment. Within patients who underwent resection of metastases, resection of other metastases than the peritoneum was also included.

### Statistical analysis

Proportion of frequencies was presented for patients with PM-CUP. Baseline characteristics of patients with isolated PM-CUP were compared to patients with PM-CUP and concurrent systemic metastases of unknown origin by means of the Chi-square test for categorical variables or unpaired T-test for continuous variables. All tests were two-sided and a *p*-value of < *0.05* was considered statistically significant. Treatments were compared between the different histological subtypes in patients with PM-CUP by means of the Chi-square test.

Median overall survival (OS) was calculated using the Kaplan–Meier method for all patients with cancer of unknown origin and for patients with PM-CUP between the different histological subtypes. Significant differences in OS were assessed by using the Log-rank test. OS was calculated from time of diagnosis until death or loss to follow-up. All patients alive on January 31, 2020, were censored. All analyses were performed with SAS statistical software (SAS system 9.4, SAS Institute, Cary, NC, USA).

## Results

### Study population

**Fig. 1:**
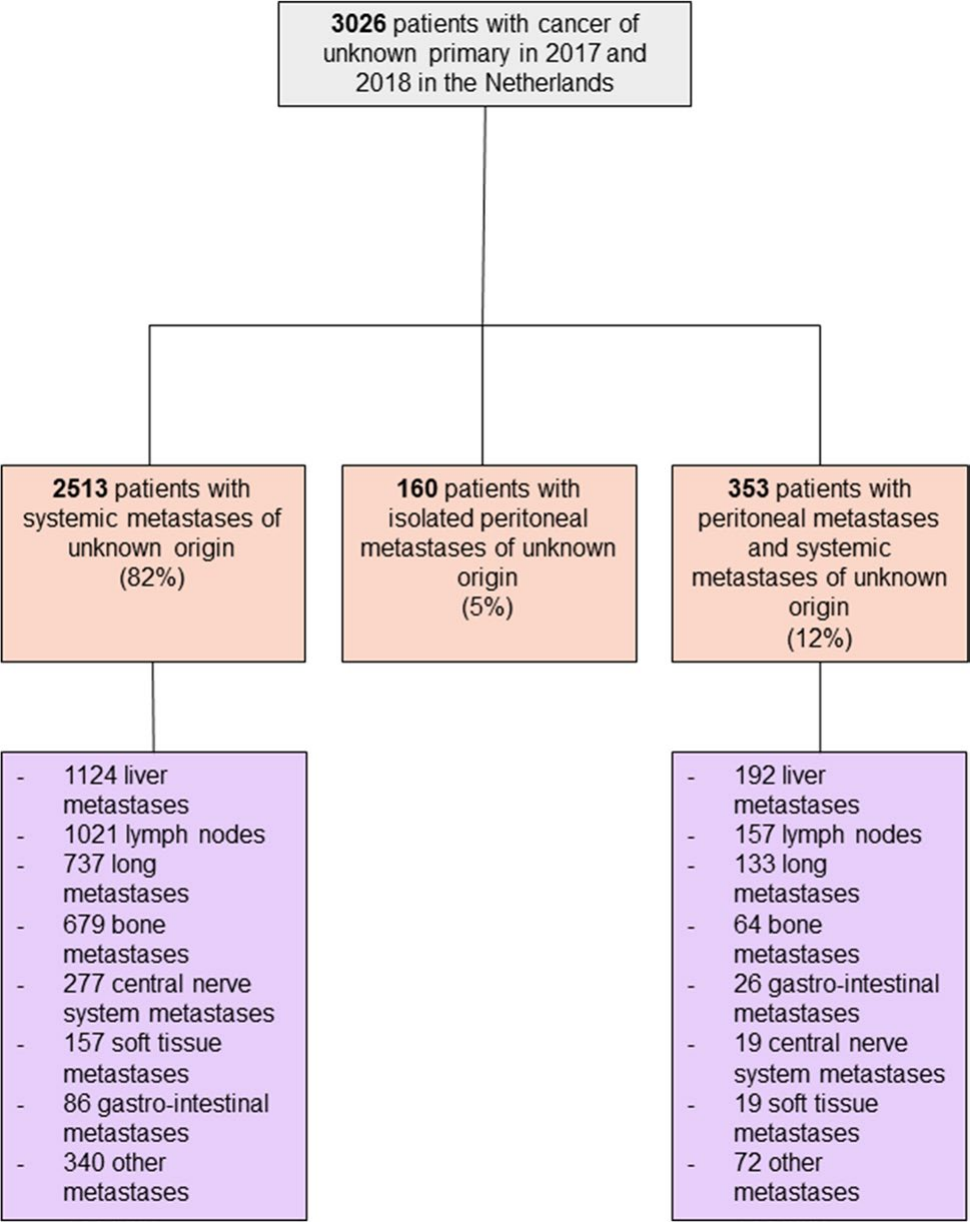
Flowchart of the study population

In 2017 and 2018, 3026 patients were diagnosed with cancer from an unknown primary origin. Among them, 513 (17%) patients had PM-CUP of which 160 (31%) presented with isolated PM-CUP and 353 (69%) presented with PM-CUP and concurrent systemic metastases of unknown origin (Fig. 1). In patients where the primary tumor location is unknown, the peritoneum ranks 5^th^ as metastatic site after the liver (*n* = 1316, 43%), lymph nodes (*n* = 1178, 39%), lung (*n* = 870, 29%) and bone (*n* = 743, 25%). In patients with PM-CUP and concurrent systemic metastases of unknown origin (*n* = 353), in 156 patients (44%) 2 organs were involved (peritoneum and one other site), in 101 patients (29%) 3 organs were involved, in 67 patients (19%) 4 organs were involved, in 29 patients (8%) > 5 organs were involved. Baseline characteristics of PM-CUP patients are presented in Table 1. Patients with isolated PM-CUP were older and had more often a (mucinous) adenocarcinoma compared to patients with PM-CUP and concurrent systemic metastases of unknown origin.

**Table 1:** Characteristics of patients with peritoneal metastases of unknown origin

	Total PM-CUP	Isolated PM-CUP	PM-CUP and systemic metastases	
	*n* = *513*	*n* = *160*	*n* = *353*	*P value*
Sex, *n* (%)				
Male	227 (44)	70 (44)	157 (44)	
Female	286 (56)	90 (56)	196 (56)	*0.878*
Age, *median (IQR)*	74 (66–82)	78 (71–85)	72 (64–80)	< *0.001*
Tumor histology, *n* (%)				
Adenocarcinoma	233 (45)	93 (58)	140 (40)	
Mucinous adenocarcinoma	22 (4)	14 (9)	8 (2)	
Carcinoid	16 (3)	1 (1)	15 (4)	
Unspecified carcinoma	185 (36)	42 (26)	143 (41)	
Other	57 (11)	10 (6)	47 (13)	< *0.001*

Percentages might not add up due to rounding; PM-CUP indicates peritoneal metastases of unknown origin; IQR indicates interquartile range

### Treatments in PM-CUP

**Fig. 2:**
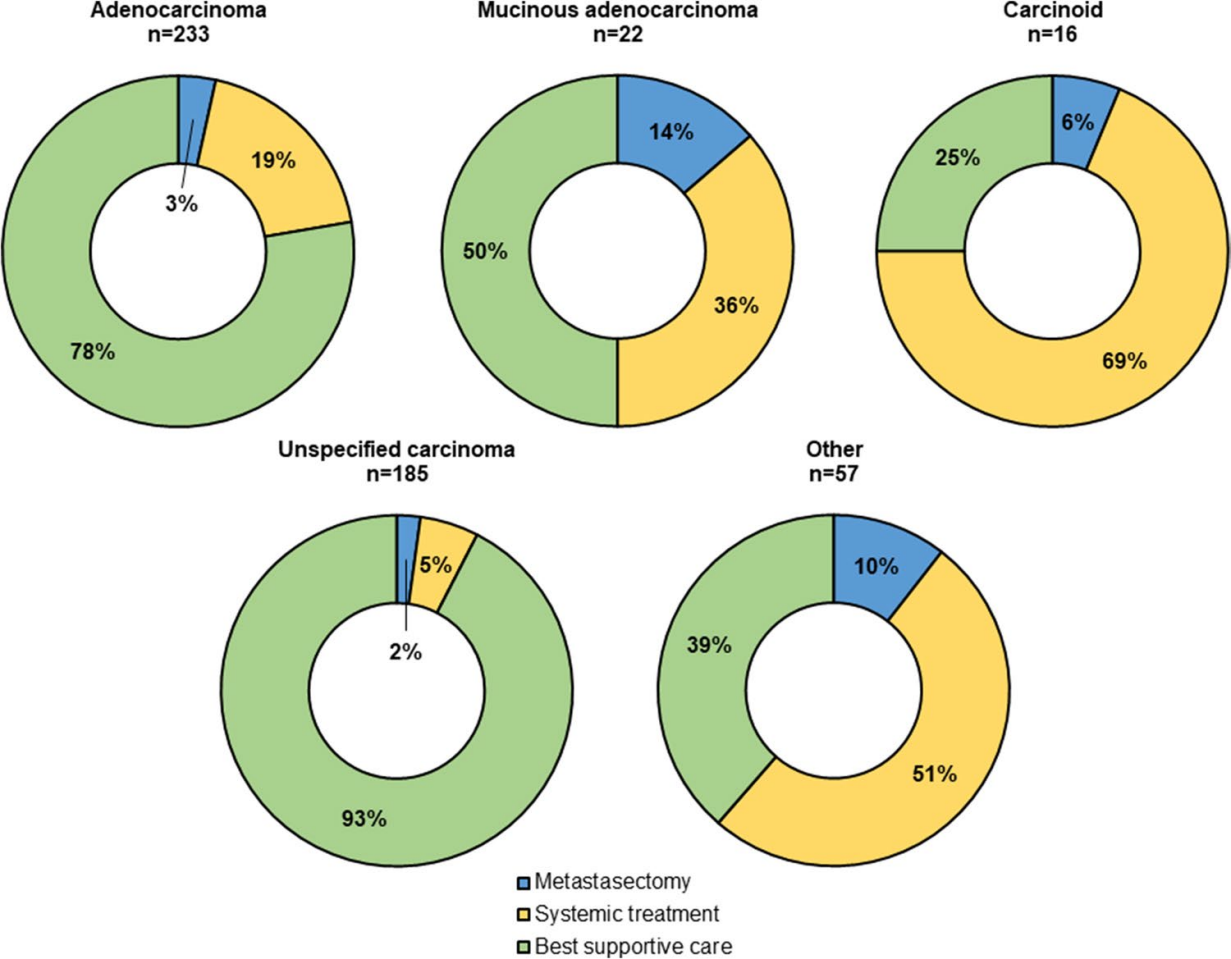
Treatments for patients with peritoneal metastases of unknown origin between the different histological subtypes

Of all PM-CUP patients, 22 (4%) patients underwent resection of metastases, 102 (20%) patients received systemic treatment and 389 (76%) patients received only BSC. In patients with isolated PM-CUP, 5 (3%) patients underwent resection of metastases, 25 (16%) patients received systemic treatment and 130 (81%) received only BSC and in patients with PM-CUP and concurrent systemic metastases of unknown origin, 17 (5%) patients underwent resection of metastases, 77 (22%) received systemic treatment and 259 (73%) patients received only BSC (*p* = *0.153*). Figure 2 provides an overview of the applied treatments between the different histological subtypes within patients with PM-CUP. Treatments differed significantly between the histological subtypes (*p* < *0.001*). Patients with mucinous adenocarcinoma more often underwent metastasectomy (14%) whereas patients with a carcinoid more often received systemic treatment (69%).

### Survival of PM-CUP

**Fig. 3:**
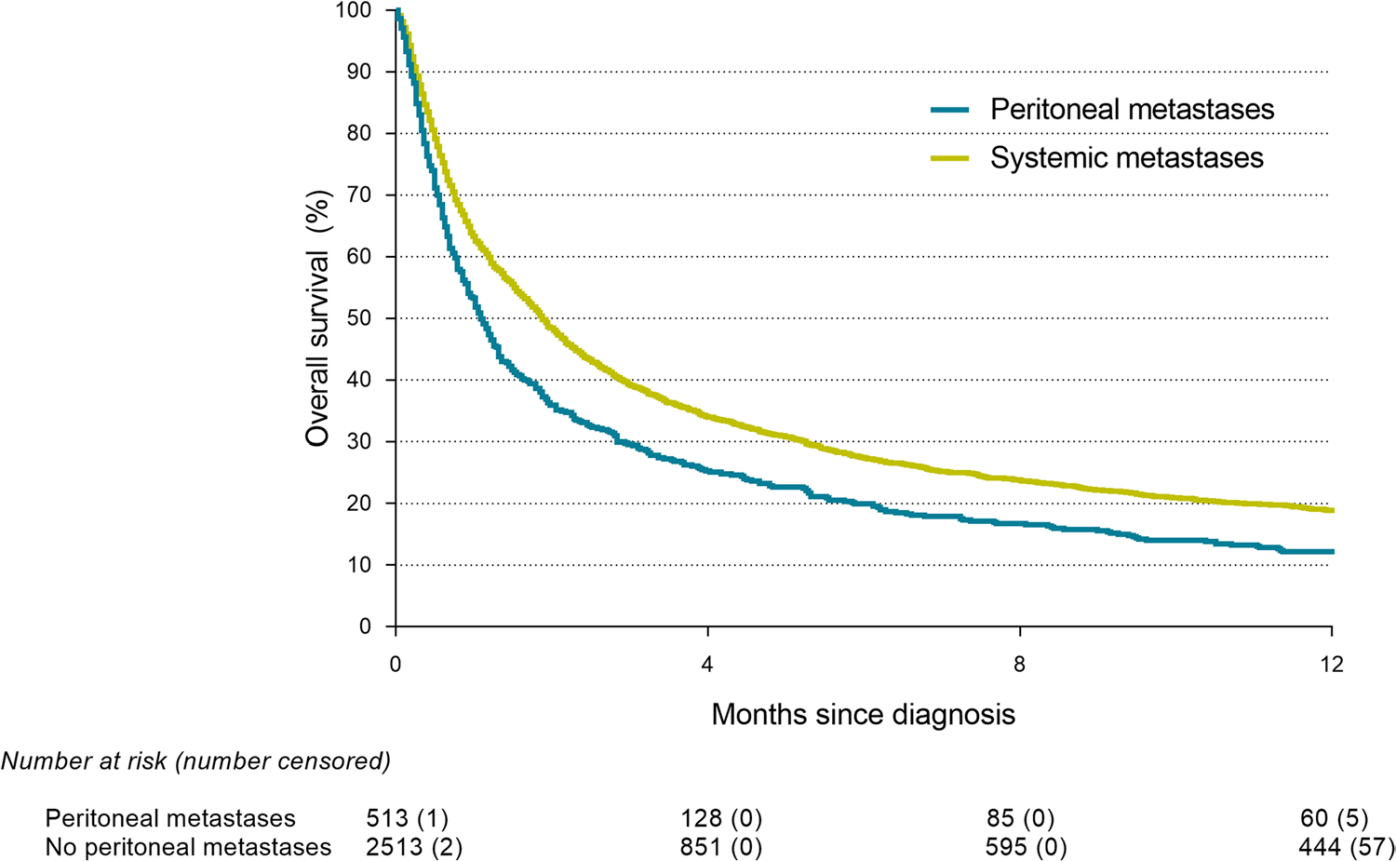
Survival of patients with cancer of unknown primary (Log-rank: *p* < *0.001*)

**Fig. 4:**
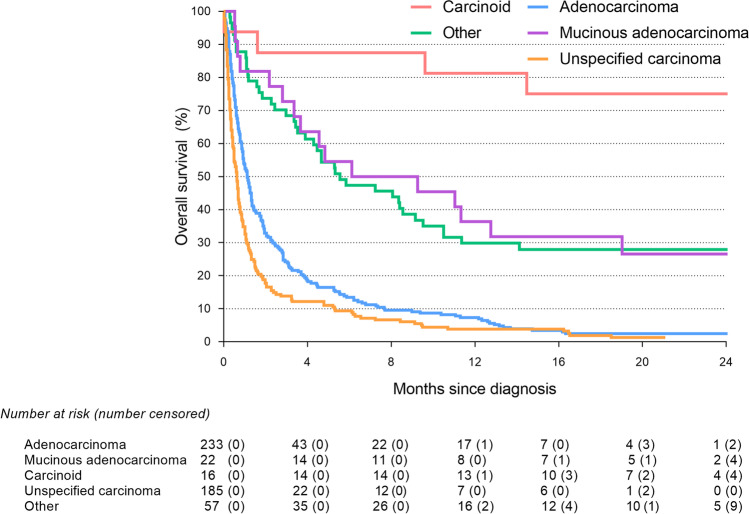
Survival of patients with peritoneal metastases of unknown primary between the different histological subtypes (Log-rank: *p* < *0.001*)

Median follow-up time in patients with PM-CUP was 4.2 months. Patients with PM-CUP had a significantly shorter OS (1.1 months, interquartile range [IQR] 0.4–4.0) as compared to patients with cancer of unknown origin without peritoneal involvement (1.9 months, IQR 0.6–7.1) (*p* < *0.001*) (Fig. 3). Median OS did not differ significantly between the patients with isolated PM-CUP (1.1 months, IQR 0.5–3.8) as compared to patients with PM-CUP and concurrent systemic metastases of unknown origin (1.1 months, IQR 0.4–4.2) (*p* = *0.712*). Median OS was significantly better in PM-CUP patients undergoing metastasectomy (8.2 months, IQR 3.9-*not reached*) or receiving systemic treatment (8.7 months, IQR 3.6–19.0) as compared to PM-CUP patients receiving only BSC (0.7 months, IQR 0.3–1.5) (*p* < *0.001*). Median OS was 1.1 months (IQR 0.5–2.8) for PM-CUP patients with an adenocarcinoma, 7.7 months (IQR 2.8-*not reached*) for PM-CUP patients with a mucinous adenocarcinoma, 30.5 months (IQR 22.4-*not reached*) for PM-CUP patients with a carcinoid and 0.6 months (IQR 0.3–1.3) for PM-CUP patients with an unspecified carcinoma (*p* < *0.001*) (Fig. 4).

## Discussion

The present study showed that PM-CUP was diagnosed in 17% of all patients with an unknown primary tumor. Among these patients, 31% patients presented with isolated PM-CUP. To date, this is the highest reported incidence of PM-CUP in population-based studies [5, 6, 17].

We have previously shown that PM-CUP were diagnosed in 11% of all patients with an unknown primary tumor in a cohort diagnosed from 1984 and upwards. Previously published population-based studies reported an incidence of PM-CUP ranging from 9 to 13%, with 2012 as the most recent reported year [5, 6, 17]. Interestingly, these reported incidence rates of PM-CUP were lower than in the incidence of 17% in the present study. Meanwhile, recent literature showed that the incidence of cancer from an unknown primary in general decreases due to the improvement and increased use of diagnostic tools such as positron emission tomography (PET)–computed tomography (CT) or more extensive morphological examination and therefore more effective detection of the primary tumor [5, 18]. The increasing incidence of PM-CUP in this study could be a relative increase due to an overall decrease of patients with cancer of unknown primary. This could be caused by a lack of further diagnostic testing for a primary tumor due to the dismal prognosis of patients in whom PM-CUP are present, in contrast to patients with metastases where the suspected prognosis warrants further investigation.

Patients with PM-CUP have a dismal prognosis with a median OS of 1.1 months as shown in this study. This is comparable to the median OS of 42 days in PM-CUP patients reported in our previously published cohort [5]. This implies that limited progress, on improving the prognosis of these patients, has been made in the past decade.

A finding with clinical importance is that patients with PM-CUP with a carcinoid histology had a remarkably higher survival than patients with other histological subtypes. Previous studies also reported that neuroendocrine carcinomas (e.g., carcinoid) of unknown primary in general have a more prognostic favorable clinicopathological entity as compared to other metastases of unknown primary [19]. This is partly explained by the inherently less aggressive behavior of neuroendocrine tumors as well as the availability of an effective systemic treatment [20]. Indeed, in the current cohort the proportion of patients receiving systemic treatment is relatively high in PM-CUP patients with a carcinoid as compared to the other histological types. Not surprisingly, the treatment in these patients consisted predominantly of hormone therapy, such as octreotide. One has to realize however that according to a population-based study on neuroendocrine carcinomas, the survival of neuroendocrine carcinomas of unknown primary was worse than those with an identified primary tumor [21]. This is probably because metastases of unknown primary in general are often characterized by a more aggressive tumor behavior [22].

In the present study, almost 80% of the patients with PM-CUP did not receive any treatment. An explanation for this remarkably high number could be that half of the patients already died within the first month after the diagnosis. Consequently, these patients did not have the opportunity to start with any form of treatment. In a previous report, we showed that 87% of the patients did not receive any treatment. In this cohort, upward from 1984, the usage of systemic therapy was increased from 8% in the earliest period to 16% in most recent years (2010) [5]. In our present cohort, 20% of all patients received systemic treatment, which empowers this previous reported increasing trend in systemic treatment application for patients with PM-CUP. Nevertheless, survival did not improve in this period of time despite this increasing trend in systemic treatment application.

Patients who received tumor-directed treatment in the current study had a significantly better OS compared to patients who did not receive any treatment. However, these reported outcomes should be interpreted with care because it is conceivable that treatment selection bias might play an important role, as patients with a good condition are more likely to receive tumor-directed treatment [23]. Furthermore, especially in patients with cancer of unknown primary, performance status appeared to be an important prognostic factor for survival. Therefore, according to the Dutch guidelines, it is recommended to make a distinction between patients with a low performance status and a good performance whether to receive tumor-directed treatment or not. Unfortunately, in the present study, data on performance status was missing in a substantial number of patients and therefore, possible influence on the given treatments could not be investigated.

As previously stated, new multimodal treatment strategies for peritoneal malignancies have changed the prognosis of patients with PM from a variety of origins. In patient with isolated and limited colorectal PM, survival was significantly better in patients undergoing CRS-HIPEC, and therefore this treatment strategy is now recommended by most (inter)national guidelines [10, 24, 25]. Furthermore, promising results have been published in studies on CRS-HIPEC for strictly selected patients with PM from ovarian cancer and currently a randomized controlled trial (PERISCOPE II, NCT03348150) investigates the role of this treatment modality in patients with isolated limited gastric PM [9, 11]. Besides new treatment options with curative intent, different variants of intraperitoneal chemotherapy are currently being explored for patients with more extensive disease [12, 13, 15, 16]. Hence, as effective treatment options are becoming more available for patients with peritoneal malignancies, it is crucial that the primary tumor will be identified. However, despite the recommendation in the Dutch guidelines for a high dose CT or PET/CT scan in patients with an unknown primary tumor, a recent study demonstrated that only 25% of these patients received this extensive diagnostic work-up [23]. This clearly needs more attention in daily clinical practice.

This study has some limitations. First, data on performance status were missing in a substantial amount of patients and therefore possible influence of this factor could not be investigated. Second, there was no data available about the use of diagnostic tools in PM-CUP patients. However, this study used nationwide data from the NCR which provides highly accurate data on tumor and patients characteristics, strengthening the generalizability.

## Conclusions

This study provides an up-to-date overview of the incidence of patients with PM-CUP. PM-CUP were diagnosed in 17% of all patients with cancer of unknown primary and the survival of all patients with PM-CUP was extremely poor. Moreover, in comparison with our previous reported cohort, a continuous increasing trend in the application of systemic treatment has been shown. Nevertheless, this increasing trend did not result in better survival outcomes for patients with PM-CUP. Despite this, survival differed among each histological subtype and recently more treatment options became available for a selected group of patients with peritoneal malignancies. Therefore, it is of great importance to identify the histology of the PM and the primary tumor whenever possible.
